# The Celiac Disease Patients' Ability to Experience Pleasure

**DOI:** 10.1155/2019/2030751

**Published:** 2019-02-28

**Authors:** Fabiana Zingone, Monica Siniscalchi, Luna Carpinelli, Paola Iovino, Letizia Zingone, Carolina Ciacci

**Affiliations:** ^1^Department of Surgery, Oncology and Gastroenterology, University of Padua, Padua, Italy; ^2^Celiac Center, AOU San Giovanni di Dio e Ruggi di Aragona, University of Salerno, Department of Medicine, Surgery, and Dentistry Scuola Medica Salernitana, Salerno, Italy

## Abstract

The motivation or ability to experience pleasure has been scarcely studied in celiac disease (CeD). We aimed to investigate the hedonistic feelings/anhedonia and sexual pleasure in CeD patients on a gluten-free diet (GFD) compared to controls. We recruited adult CeD patients at follow-up consecutively visited from April 2017 to April 2018 and controls from the hospital staff and friends of CeD patients. Participants completed the Snaith–Hamilton Pleasure Scale, measuring the levels of anhedonia, and answered three questions about physical contact, sexual activity, and modification of their life on a GFD. We included 178 CeD patients and 173 healthy controls. Seventeen patients (9.5%) and fourteen controls (8.1%) had anhedonia. We did not find any correlation between the presence of anhedonia and the length in years of GFD neither with the dietary compliance and age at the test. 10.7% patients and 8.7% controls reported of not having pleasure in physical contact and 5.06% CeD and 3.5% controls in feeling attraction for another person; 36.56% said a worsening of their life on a GFD. Our results show that CeD patients on a GFD are similar to controls in anhedonia and sexual problems, despite one-third reported a worsening of their life.

## 1. Background

The vast majority of people, at some point in their life, lose interest in things that used to motivate them. However, there is a condition, anhedonia, in which it becomes impossible to experience pleasure from things that once elicited excitement, such as music, sex, food, and company with friends. The term “anhedonia” is traditionally used to refer a specific psychopathological condition characterized by a deficit of ability to experience pleasure in activities and situations usually considered gratifying [1, 2].

The most recent psychopathological literature on pleasure shows us that two main components compose the hedonic capacity: *an anticipatory one* (“I want,” wanting) and *a consumer one* (“I like,” liking) [3–5]. The anticipatory pleasure is a pleasure closely linked to motivation and future activities, while the consumption is closely related to satisfaction, to the fulfillment of a desire, and to the experience concluded at a precise moment and in response to a specific positive stimulus [6]. Preclinical studies of neurobiology have confirmed this distinction by correlating the two types of pleasure to different neurotransmitters and neural circuits, in particular, dopamine for anticipatory pleasure and opioid neuropeptides for consumer use [7].

Some recent experimental researches seem to accredit the hypothesis of anhedonia as a specific symptom of status depression, correlated frequently with the presence of psychomotor delay [8], suicidal ideation [9], and a high probability of the implementation of suicide behaviors [10]. Myerson [11], for the first time, considered anhedonia as a premorbid stroke of the predepressive personality, hypothesizing the existence of “hypoedonian individuals by nature,” in which the hereditary character of such constitution could be increased by several factors stressful, such as organic brain damage, childhood trauma, or unfavorable changes to the surrounding environment. According to the author, this “constitutional anhedonia” constituted, already in itself, a sort of mild form and chronic depression, which predisposed to the development of more severe clinical pictures of melancholia.

The DSM-5 criteria for major depression include both depressive and anhedonic symptoms [12]. Nevertheless, some patients with chronic diseases fulfilling the criteria for major depression can have anhedonia but the not depressed mood, a condition also called “depression without depression” [13].

Celiac disease (CeD) has been linked to the decreased quality of life and while some psychological aspects improve within a few months after starting a gluten-free diet, some patients continue to suffer from significant mental morbidity [14–17]. However, the motivation or ability to experience pleasure [18] has been scarcely studied in CeD, being the observations mainly limited to sexual satisfaction [19, 20]. To the best of our knowledge, the presence of anhedonia has never been studied in gastrointestinal disorders while it is mainly evaluated in patients with neurological disorders [21, 22].

We aimed to investigate the hedonistic feelings/anhedonia and sexual pleasure in patients with CeD on a gluten-free diet (GFD) compared to healthy subjects.

## 2. Methods

The study population consisted of adult CeD patients at follow-up on a GFD, consecutively recruited from April 2017 to April 2018 at celiac outpatients clinic of the University of Salerno and volunteers recruited from the hospital staff and friends of CeD patients. Patients underwent a brief structured interview with the psychologists on the team to exclude those with major psychiatric disturbances. Questionnaires were administered in the morning of the routine visit during the follow-up period. None of the patients refused to answer this questionnaire. For all CeD patients, the diagnosis was based on the presence of antitransglutaminase IgA and antiendomysium antibodies and an intestinal biopsy compatible with gluten-related damage. The control group consisted of individuals who were tested and found negative for serum markers of coeliac disease.

Data were collected on age, gender, marital status, and job, for CeD group years of GFD. The inclusion criteria of the study cohort were as follows: GFD from at least one year, written informed consent, age 18–60 years, and absence of major psychiatric disease. Following a psychological interview to assess the presence of mood disorders, participants completed the Snaith–Hamilton Pleasure Scale (SHAPS), composed of 14 questions measuring the levels of anhedonia [23, 24]. A total score ≥ three was considered pathological. Moreover, all subjects answered the following questions: “*I feel pleasure in such physical contact as hugs and caresses*” and “*I feel pleasure in sexual activity*” exploring sexual pleasure. They were also asked whether the CeD diagnosis had changed their life (worse/equal/better). A dietetic interview evaluated dietary compliance and self-reported by marking a score in a VAS from 0 to 10.

## 3. Statistical Analysis

Categorical and continuous variables were expressed as frequency and mean ± standard deviation or median with maximum and minimum values. Differences in frequencies were calculated using the chi-square test. Differences in continuous variables were calculated using the Student *t*-test. We used the Spearman's rank correlation coefficient (95% CI) to study the correlations between total anhedonia score and age, gluten-free diet, and dietary compliance.

## 4. Results

We included 178 CeD patients (mean age 37 ± 9.7 years, 75.3% females), on a GFD from a mean of 9.2 ± 7.3 years with average compliance of 10 and 173 healthy controls (mean age 36.1 ± 10.8 years, 66.5% females). No differences in age, sex, marital status, and job were found between the two groups (Table 1). The psychological interview did not detect any mood disorders in CeD patients.

We found a mean score of the SHAPS scale of 0.99 ± 1.3 in CeD patients and 0.7 ± 1.2 in healthy controls (*p* = 0.6). Seventeen patients (9.5%) and fourteen controls (8.1%) had anhedonia (≥3) (Table 2). Among CeD population, one patient with anhedonia reported dietary compliance of 8, two of 9, and fourteen of 10. More CeD patients than controls disagree or strongly disagree with three questions of the questionnaire: *I would enjoy my favourite television or radio program* (18.5% vs. 9.8%), *I would be able to enjoy my favourite meal* (7.9% vs. 2.3%), and *I would find pleasure in the scent of flowers or the smell of a fresh sea breeze or freshly baked bread* (7.3% vs. 1.3%). The Cronbach alpha was 0.52 for CeD and 0.56 for controls.

We did not find any correlation between the presence of anhedonia and the length in years of GFD neither with the dietary compliance and age at the test. Nineteen patients (10.7%) and fifteen controls (8.7%) reported of not having pleasure in physical contacts (*p* = 0.4) and 9 CeD (5.06%) and 6 controls (3.5%) in feeling attraction for another person (*p* = 0.5). Finally, 65 patients reported the worsening of their life after CeD diagnosis (36.52%) (Table 3).

## 5. Discussion

Anhedonia is a core symptom of major depressive disorders and schizophrenia [2]. However, some findings support the view that anhedonia is a construct that is distinct and separate from depression [25–27]. From the psychobiological point of view, anhedonia may (putatively) rely on the dopaminergic, mesolimbic, and mesocortical reward circuit, which involves the ventral tegmental area, the ventral striatum, and part of the prefrontal cortex [28, 29]. The mesocortical reward system attempts to regulate and control behavior by inducing pleasurable effects through the dopaminergic mesolimbic and mesocortical pathways. Of these pathways, the *mesolimbic pathway* plays the primary role and is associated with associative learning, reward motivation, and reinforcement. The mesocortical pathway is strongly associated with working memory, attention, and inhibitory control [7].

Our data suggest that the presence of anhedonia or compromised sexual pleasure in patients with CeD on a GFD is similar to that of the general population. The datum is relevant as it confirms that also in CeD hedonistic aspects of behavior follow separate neurologic routes of depression and brain impairment. In fact, both depressive mood [30, 31] and the so-called “silent neurological damage” of CeD that causes the subtle impairments to memory, attention, decision-making, and the speed of cognitive processing collectively referred to as “brain fog” are frequently reported by patients on GFD who are inadvertently exposed to gluten [32–34]. Nevertheless, the analysis of each item of the questionnaire showed that CeD patients seem not to be able to enjoy their favourite food and this apparently can be directly correlated with their disease and the limitation in their diet.

Our results are in line with the previous data on sexual pleasure; Ciacci et al. reported in fact that untreated CeD patients with a classical presentation had a significantly lower frequency of intercourse and a lower prevalence of individuals satisfied with their sexual life patients compared to healthy controls while patients with subclinical coeliac disease did not show significant differences for any indices of sexual behavior. Moreover, considering CeD patients after one year of treatment had improved values for all indices of sexual conduct. A direct correlation with the clinical presentation was also found in 2016; the authors described that partner burden is directly correlated with patient symptom severity and it increases with poorer sexual and relationship satisfaction [19]. CeD patients of the present study are on GFD, and only about 40% of them experienced symptoms before diagnosis.

The main limitation of this study is the lack of a longitudinal design that could have evaluated the presence of anhedonia at diagnosis and its improvement on a GFD. Moreover, we have not looked for very mild/mild mood alteration other than clinical relevant depression and/or anxiety which are common in CeD disease and also on GFD, and which can indirectly influence the presence of anhedonia. However, our patients answered the questions of whether the CeD diagnosis had changed their life and 36.52% reported a worsening of their life. This should take into account and accurately evaluated during the CeD follow-up. However, also the deterioration of the quality of life does not seem to influence the presence of anhedonia.

In conclusion, our results show that CeD patients on a GFD are similar to nonceliac controls and do not have sexual problems. Our data demonstrate for the first time at our best knowledge that CeD patients can feel pleased with the most common experience of life. However, future studies measuring the different components of anhedonia must be done as well as other important affective deficit as alexithymia should be studied. Finally, a similar analysis should be done at the time of diagnosis to show the influence of the diet of these aspects.

## Figures and Tables

**Table 1:** Study population characteristics.

Variables			*P*
*N*	178 CeD subjects	173 Controls	
Sex (% females)	134 (75.3)	115 (66.5)	0.07
Age test	37 ± 9.79	36.09 ± 10.84	0.7
Job			
(i) Unemployed	7 (3.9)	6 (3.5)	0.06
(ii) Student	30 (16.8)	28 (16.2)	
(iii) Stay-at-home	20 (11.2)	12 (6.9)	
(iv) White collar workers	74 (41.6)	63 (36.4)	
(v) Professional	40 (22.5)	42 (24.3)	
(vi) Blue collar workers	7 (3.9)	22 (12.72)	
Relationship			
(i) Single	26 (14.6)	36 (20.8)	0.7
(ii) In a relationship	58 (32.6)	64 (36.9)	
(iii) Married	94 (52.8)	73 (42.2)	
Time of GFD	9.32 ± 7.3 Median 8 (range 1-39)	—	
Compliance^∗^	9.6 ± 1.1 Median 10 (range 0-10)	—	

^∗^1 patient reported 0, three reported 5, 1 said seven, 9 reported 8, 24 reported 24, and all the rest 10.

**Table 2:** The SHAPS questionnaire.

	CeD patients (*N* = 178)	Controls (*N* = 173)	*P*
	Disagree or strongly disagree (*N* (%))	Disagree or strongly disagree (*N* (%))	
*I would enjoy my favourite television or radio program*	*33 (18.5)*	*17 (9.8)*	*0.02*
I would enjoy being with family or close friends	2 (1.12)	1 (0.58)	0.57
I would find pleasure in my hobbies and pastimes	8 (4.5)	6 (3.5)	0.62
*I would be able to enjoy my favourite meal*	*14 (7.9)*	*4 (2.31)*	*0.02*
I would enjoy a warm bath or refreshing shower	5 (2.8)	2 (1.16)	0.26
*I would find pleasure in the scent of flowers or the smell of a fresh sea breeze or freshly baked bread*	*13 (7.3)*	*3 (1.73)*	*0.01*
I would enjoy seeing other people's smiling faces	2 (1.12)	7 (4.05)	0.08
I would enjoy looking smart when I have made an effort with my appearance	10 (5.62)	9 (5.2)	0.86
I would enjoy reading a book, magazine or newspaper	38 (21.3)	27 (15.6)	0.16
I would enjoy a cup of tea or coffee or my favourite drink	10 (5.6)	11 (6.3)	0.77
I would find pleasure in small things; e.g., bright sunny day, a telephone call from a friend	4 (2.25)	7 (4.05)	0.3
I would be able to enjoy a beautiful landscape or view	4 (2.25)	5 (2.9)	0.7
I would get pleasure from helping others	32 (17.9)	29 (16.7)	0.7
I would feel pleasure when I receive praise from other people	2 (1.12)	1 (0.6)	0.57
Number of subject with the pathological score (>3)	**17 (9.55)**	**14 (8.09)**	**0.63**
Mean score	0.99 ± 1.3	0.7 ± 1.2	**0.6**

**Table 3:** The other questions included in the survey.

	CeD patients	Controls	*P*
Pleasure in having such physical contacts as hugs and caresses	19 (10.7)	15 (8.7)	0.5
Pleasure in feeling physical attraction for another person	9 (5.06)	6 (3.5)	0.46
Celiac disease diagnosis changes my life?			
(i) No changes	77 (43.26)		
(ii) It is worse	65 (36.52)		
(iii) It is better	36 (20.22)		

## Data Availability

The data used to support the findings of this study are available from the corresponding author upon request.
